# The Memory Benefit to Aged APP/PS1 Mice from Long-Term Intranasal Treatment of Low-Dose THC

**DOI:** 10.3390/ijms23084253

**Published:** 2022-04-12

**Authors:** Oksana Fihurka, Yuzhu Hong, Jiyu Yan, Breanna Brown, Xiaoyang Lin, Ning Shen, Yanhong Wang, Haohan Zhao, Marcia N. Gordon, David Morgan, Qingyu Zhou, Ping Chang, Chuanhai Cao

**Affiliations:** 1 Department of Pharmaceutical Sciences, Taneja College of Pharmacy, University of South Florida, Tampa, FL 33612, USA; yuzhu2018@gmail.com (Y.H.); jiyu_yan12@yahoo.com (J.Y.); breannabrown@knights.ucf.edu (B.B.); xlin3@usf.edu (X.L.); ningshen@usf.edu (N.S.); yanhongwang@usf.edu (Y.W.); haohan@usf.edu (H.Z.); qzhou1@usf.edu (Q.Z.); 2 Department of Neurology, College of Medicine, University of South Florida, Tampa, FL 33612, USA; 3 Department of Chemistry, College of Arts & Sciences, University of South Florida, Tampa, FL 33620, USA; 4 Department of Translational Neuroscience, College of Human Medicine, Michigan State University, Grand Rapids, MI 49503, USA; mngordon@msu.edu (M.N.G.); scientist.dave@gmail.com (D.M.); 5 Rhodes Pharmaceuticals L.P., Coventry, RI 02816, USA; ping_wu_chang@sbcglodl.net; 6 USF-Health Byrd Alzheimer Institute, Tampa, FL 33613, USA

**Keywords:** THC, APP/PS1, amyloid beta, p-tau

## Abstract

THC has been used as a promising treatment approach for neurological disorders, but the highly psychoactive effects have largely warned off many scientists from pursuing it further. We conducted an intranasal treatment using low-dose THC on 12-month-old APP/PS1 mice daily for 3 months to overcome any potential psychoactive response induced by the systemic delivery. Our results demonstrate that the THC nasal treatment at 0.002 and 0.02 mg/kg significantly slowed the memory decline compared to that in the vehicle-treated transgenic mouse control group. An enzyme-linked immunosorbent assay showed that the Aβ1–40 and 1–42 peptides decreased in the THC-treated groups. The Western blot data indicate that long-term low-dose THC intranasal administration promoted p-tau level reduction and mitochondrial function marker redistribution. The blood biochemical parameter data demonstrate some insignificant changes in cytokine, immunoglobulin, and immune cell profiles during intranasal THC treatment. Intranasal delivery is a non-invasive and convenient method that rapidly targets therapeutics to the brain, minimizing systemic exposure to avoid unwanted adverse effects. Our study provides new insights into the role of low-dose THC intranasal treatment as a pharmacological strategy to counteract alterations in Alzheimer’s disease-related cognitive performance.

## 1. Introduction

Alzheimer’s disease (AD) is a multifactorial mental illness characterized by age-dependent memory loss, ultimately leading to a progressive decline in cognitive function. AD accounts for 60–80% of dementia cases, making it one of the leading forms of dementia. One of the primary neuropathological hallmarks of AD is the accumulation of products of amyloid beta precursor protein (APP) degradation, leading to β-amyloid peptide (Aβ) accumulation in plaques in the hippocampal and cortical areas of the brain, important for memory and cognition [1]. Hence, Aβ in its two most common isoforms, Aβ1–40 and Aβ1–42, provides a potential target to treat AD, with the more hydrophobic, fibrillogenic, and toxic Aβ1–42 being the principal species found in the brain lysates of patients with AD [2,3]. The abundance and solubility of Aβ peptides are critical determinants of the formation of amyloid plaques as well as the onset and progression of AD. A second distinctive feature of AD is a disruption of the microtubule network, caused by hyperphosphorylated microtubule-associated protein (p-tau) aggregates inside the neurons, which disrupt the cells’ ability to strengthen their connections with other neurons, preventing them from stabilizing new memories [4,5]. Aβ is upstream of tau in AD pathogenesis and triggers the conversion of tau from a normal to a toxic (phosphorylated) state, but there is also evidence that toxic tau enhances Aβ toxicity via a feedback loop [6]. Moreover, direct responses to Aβ and tau deposition are inflammation and a decline in the immune system, which also play very important roles in AD development [7], by the stimulation of microgliosis and astrogliosis, mitochondrial dysfunction, oxidative stress, excitotoxicity, etc., with concomitant loss of synapses and neurons, a progressive decline in cognitive performance, and robust memory loss. 

Currently, AD presents one of the world’s biggest healthcare issues. Unfortunately, present AD treatments do not prevent, stop, or reverse disease progression, highlighting the need for new, more effective therapeutics and treatment approaches. The discovery of a link between the endocannabinoid system (ECS) and AD has provided a new therapeutic target for the treatment of patients suffering from AD. Studies have demonstrated the ability of cannabinoids (tetrahydrocannabinol (THC) and cannabidiol (CBD)) to serve as signaling molecules that regulate downstream events implicated in AD pathology [8,9]. The ECS is a nerve signaling system throughout the human body that helps maintain physiological, emotional, and cognitive stability. It is also a part of the anti-aging homeostatic defense system. In fact, THC and CBD have a strong affinity for endogenous cannabinoid receptors (CB_1_ and CB_2_), but THC, unlike CBD, has psychoactive properties. Research studies demonstrate that memory impairment is due to the use of high THC doses. Paradoxically, low THC doses have the capacity to rescue old mice’s brains and prevent age-related cognitive impairments [10]. In fact, THC modulates memory and cognition in a biphasic and age-dependent manner: in old animals, low concentrations improve memory and cognition, while high concentrations impair these functions; in young animals, even a low concentration is detrimental [11]. In agreement with this fact, recent studies have demonstrated that low-dose THC promotes hippocampal neurogenesis; slows down AD progression by reducing the Aβ level; prevents neurodegenerative processes from occurring in animal models of AD; protects against inflammation-induced cognitive damage; and restores memory and cognitive function in old mice [11,12]. Thus, continuous treatment with a therapeutically effective dose of cannabinoids could be a potential multifunctional therapy option for AD.

Earlier, our in vitro study demonstrated the therapeutical value of low-dose THC use for slowing the hallmark characteristics of AD. Importantly, the anti-Aβ and anti-tau effects of THC were found to increase in a time-dependent manner [13], which indicated the necessity of the transition to an animal study. A recent in vivo study exhibited the memory-improving effect of low-dose THC treatment that is associated with its inhibitory effect on Aβ aggregation, GSK3β (glycogen synthase kinase 3β) activity, and tau phosphorylation in the brain [14]. The current in vivo research aimed to study the efficacy of intranasal treatment of aged APP/PS1 mice with low-dose THC for slowing down cognitive function decline, lowering Aβ levels in the brain, inhibiting p-tau production, and improving mitochondrial function and overall AD attenuation. Among a large number of drug administration routes and methods, the intranasal route of transportation directly delivers the drugs to the brain. Importantly, intranasally administered therapeutics bypass the blood–brain barrier (BBB) and avoid the systemic exposure and side effects associated with therapeutics that enter the bloodstream [15]. In addition, the primary advantages of intranasal delivery of therapeutics to the brain are its simplicity and safety, which allow repetitive administrations for prolonged periods of time [16,17]. 

Thus, within the current study, we tested our hypothesis that continuous intranasal administration of THC in low doses effectively prevents or even reverses AD-associated memory impairments in an aged AD animal model.

## 2. Results

### 2.1. Long-Term Low-Dose THC Intranasal Treatment Protects 12-Month-Old APP/PS1 Mice from Memory Loss 

**Figure 1:**
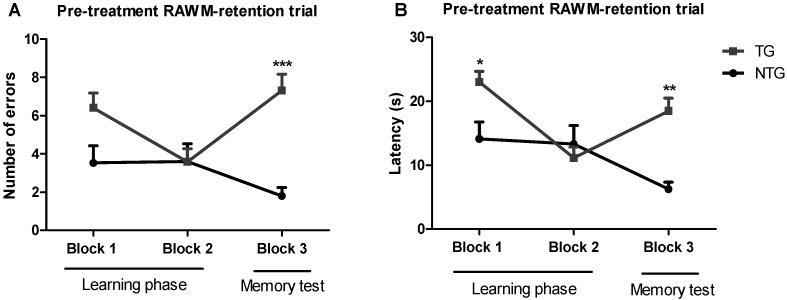
Assessment of the spatial reference memory of 12-month-old transgenic (APP/PS1) and non-transgenic mice on a radial arm water maze device. The number of errors (**A**) baseline and latency baseline (**B**) represent the difference in cognitive performance in the 5th trial of APP/PS1 transgenic mice (TG group) and non-transgenic wildtype mice (NTG group). Data of 9 days of study were grouped into 3 blocks and are expressed as the mean ± SEM (*n* = 5 per NTG group, *n* = 15 per TG group). Latency and number of errors were analyzed using a two-way ANOVA multiple comparison test. * *p* < 0.05, ** *p* < 0.01, and *** *p* < 0.001 for comparisons among NTG mice and APP/PS1 mice.

The radial arm water maze (RAWM) method was implemented to test spatial learning in 12-month-old transgenic APP/PS1 and non-transgenic mice. This method allowed measuring both working and reference memory through a reward system: in this case, escaping from the water. The non-treated 12-month-old transgenic APP/PS1 mice (TG) and non-transgenic mice (NTG) were used as controls. Training trials were conducted in blocks of five trials per day (Figure 1). The results of the baseline spatial memory evaluation show that NTG control mice rapidly learned the new platform location, resulting in a decrease in the number of errors and time spent in the wrong place (i.e., latency), as compared with the APP/PS1 mice. After spatial memory evaluation, APP/PS1 mice were randomly assigned to the vehicle group, or the 0.002 mg/kg or 0.02 mg/kg THC treatment groups. The mean latencies of the three study groups were not significantly different.

**Figure 2:**
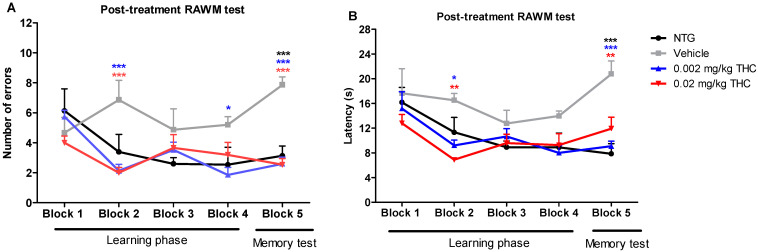
Assessment of the effect of low-dose THC intranasal treatment on the spatial reference memory of APP/PS1 mice on a RAWM device. The number of errors (**A**) and latency (**B**) represent the difference in cognitive performance of NTG and TG mice prior to treatment. Both parameters represent the difference in cognitive performance in the 5th trial of 15-month-old vehicle-treated mice, THC-treated mice at 0.002 and 0.02 mg/kg doses, APP/PS1 transgenic mice (TG group), and 15-month-old non-treated mice (NTG group). Data of 15 days of study were grouped into five blocks and are expressed as the mean ± SEM (*n* = 5 per animal group). Latency and number of errors were analyzed using a two-way ANOVA multiple comparison test. * *p* < 0.05, ** *p* < 0.01, and *** *p* < 0.001 for NTG group and 0.002 and 0.02 mg/kg THC-treated groups compared to vehicle-treated group.

Post-treatment studies were performed after the course of the intranasal treatment with the vehicle or THC at 0.002 or 0.02 mg/kg doses. Every TG mouse from the treatment groups received intranasal administration of the vehicle or THC every day for three months. Vehicle-treated 12-month-old TG and NTG mice were used as controls. The post-treatment behavioral data show that the TG mice had significant memory decline and no ability to learn. In the fifth trial, vehicle-treated TG mice demonstrated significant behavioral impairment in both the number of errors made and the time of continuing to make the wrong choices by entering the wrong arms (latency in seconds) as compared with the NTG mice (Figure 2). Both animal groups treated with THC demonstrated a gradual improvement in learning abilities and memory functions. Through the fifth trial, the TG mice receiving THC intranasally showed a significantly lower number of errors and latencies than the TG mouse control group. THC-treated APP/PS1 mice showed a remarkable improvement in latency, by spending half as much time on finding the platform and escaping from the maze. Both THC-treated animal groups made 67% less errors by the end of all trial sessions. It should be noted that both the 0.002 and 0.02 mg/kg THC doses were equally effective in memory protection. Importantly, no evidence of a significant difference in both behavior parameters was found between the NTG control mice and THC-treated APP/PS1 mice. The RAWM study demonstrated that low-dose THC effectively slowed down Alzheimer’s-associated memory decline or even restored memory functions. 

### 2.2. Continuous Low-Dose THC Intranasal Treatment Modulates Aβ Levels in Plasma and Brain Regions Responsible for Memory 

**Figure 3:**
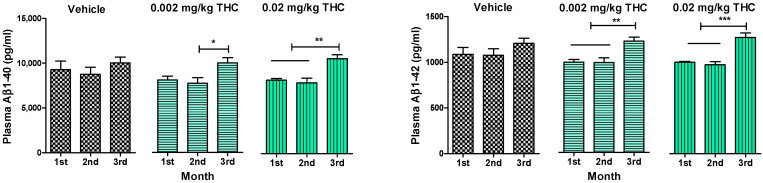
Determination of Aβ1–40 and Aβ1–42 levels in the plasma of APP/PS1 mice using ELISA. Plasma samples were collected from the APP/PS1 mice every month after beginning intranasal THC treatment. No significant difference in Aβ1–40 and Aβ1–42 levels was found among the study groups. Plasma Aβ1–40 and Aβ1–42 levels of THC-treated animals significantly increased by the 3rd month of treatment. The data are expressed as the mean ± SEM (*n* = 5 per group). * *p* < 0.05, ** *p* < 0.01, and *** *p* < 0.001. A one-way repeated measures ANOVA was used to analyze the data, followed by a Tukey post hoc multiple comparison test.

**Figure 4:**
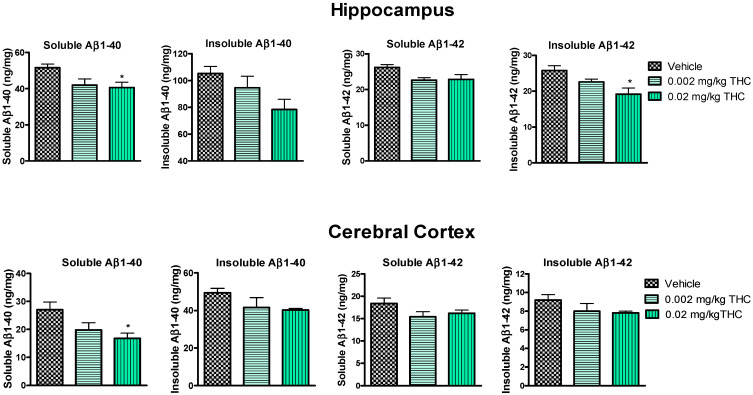
Evaluation of both soluble and insoluble Aβ1–40 and Aβ1–42 levels in the hippocampus and cerebral cortex brain tissues of APP/PS1 mice using ELISA. Tissue samples were collected from the TG mice after the 3-month THC or vehicle intranasal treatment. A significant difference in soluble Aβ1–40 was found in both brain regions, and a significant difference in insoluble Aβ1–42 was found in the hippocampus after treatment with 0.02 mg/kg THC. Brain tissue samples’ Aβ1–40 and Aβ1–42 peptide concentrations are presented in ng/mg of total protein. Data are expressed as the mean ± SEM (*n* = 5 per group). SEM is shown as error bars. * *p* < 0.05 for comparisons among vehicle-treated APP/PS1 mice and APP/PS1 mice treated with 0.002 or 0.02 mg/kg THC using one-way ANOVA followed by a Tukey post hoc multiple comparison test.

An enzyme-linked immunosorbent assay enabled us to show the changes in the levels of soluble and insoluble Aβ1–40 and Aβ1–42 peptides in plasma (Figure 3), the hippocampus, and the cerebral cortex (Figure 4). Throughout the experiment, Aβ peptide levels in plasma were tracked and measured monthly. Thus, it was found that long-term intranasal treatment with 0.002 or 0.02 mg/kg THC appeared to have a statistically significant impact on the Aβ1–40 and Aβ1–42 peptide levels at the third month of treatment (Figure 3). Notably, three months of intranasal treatment with 0.02 mg/kg THC had an exceptional influence on the Aβ plasma peptide level.

Hippocampal and cerebrocortical levels of Aβ peptides were assessed after three months of daily intranasal treatment. Overall, the treatment of either 0.002 or 0.02 mg/kg of THC had a beneficial effect on the reduction in the Aβ concentration in the brain in comparison to the vehicle-treated brain tissue samples (Figure 4). More importantly, the continuous 0.02 mg/kg THC intranasal treatment significantly lowered the amount of the soluble Aβ1–40 form in the hippocampus and cerebral cortex. Additionally, the insoluble Aβ1–42 peptide level was significantly reduced in hippocampal samples from 0.02 mg/kg THC-treated mice. The results from the brain tissue study show that THC at a dose of 0.02 mg/kg tended to decrease the soluble and insoluble Aβ1–40 and Aβ1–42 peptide levels.

### 2.3. Low-Dose THC Intranasal Treatment Has a Positive Effect on p-GSK3β/GSK3β and p-Tau/Tau Levels in the Brain

**Figure 5:**
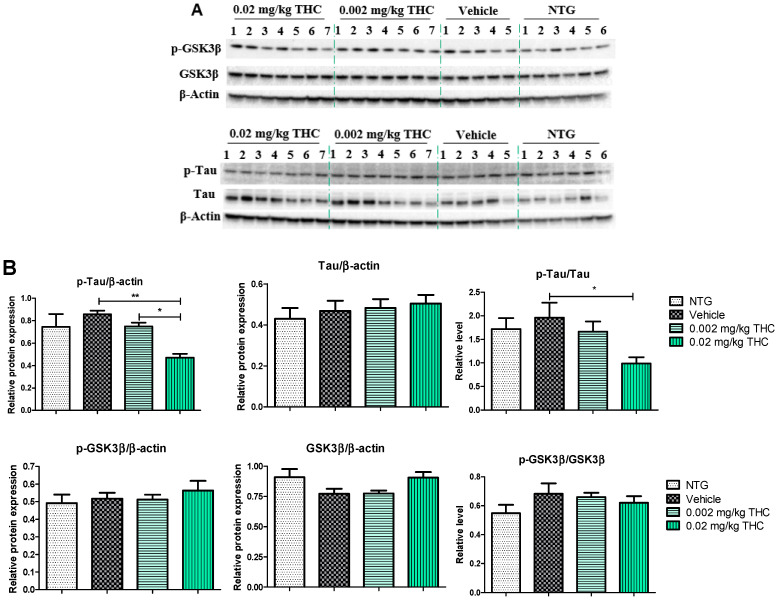
Analysis of molecular markers associated with the neuropathologic changes in AD. (**A**) Representative Western blot images of tau, p-tau, GSK3β, and p-GSK3β expression. (**B**) Tau, p-tau, GSK3β, and p-GSK3β relative protein expression levels normalized to β-actin. Tissue samples were collected from the TG mice after the 3-month THC or vehicle intranasal treatment and from NTG mice. A significant difference in the expression of p-tau was found in the experimental animal group treated with the 0.02 mg/kg THC dose. No statistically significant differences in tau, GSK3β, and p-GSK3β were observed between the vehicle and THC treatments in APP/PS1 mice. Data are presented as the mean ± SEM (*n* = 5 for the TG vehicle-treated group; *n* = 6 for the NTG group; and *n* = 7 for the 0.002 and 0.02 mg/kg THC-treated groups). SEM is shown as error bars. * *p* < 0.05, ** *p* < 0.01 for comparisons among vehicle-treated TG mice, NTG mice, and APP/PS1 mice treated with 0.002 or 0.02 mg/kg THC using one-way ANOVA followed by a Tukey post hoc multiple comparison test.

The effects of low-dose THC intranasal treatment on tau, phospho-tau (p-tau), GSK3β, and phospho-GSK3β (p-GSK3β) expression in the APP/PS1 mice were evaluated by immunoblotting analysis. The tau defect that leads to aggregation and neurodegeneration is referred to as hyperphosphorylated tau [18]. Tau phosphorylation is highly regulated via protein kinases. Numerous studies have shown that GSK3β robustly phosphorylates a broad range of substrates, including a majority of sites on tau [19]. Importantly, GSK3β phosphorylation leads to inhibition of GSK3β kinase activity and the formation of p-tau aggregates [20]. As shown in Figure 5, p-tau expression in the APP/PS1 mouse brains after the 3 months of 0.02 mg/kg THC treatment was significantly lower than in the vehicle- and 0.002 mg/kg THC-treated mouse brains. No significant difference in tau, GSK3β, and p-GSK3β expression was found between NTG mice and APP/PS1 mice treated with the vehicle or THC. Thus, prolonged 0.02 mg/kg THC intranasal treatment significantly decreased the p-tau level without affecting GSK3β.

### 2.4. THC Intranasal Treatment Improves Mitochondrial Function in APP/PS1 Mice

Mitochondria are critical for providing sufficient energy to maintain endogenous neuroprotective and reparative mechanisms, while disturbances in mitochondrial functions lead to neuronal degeneration. Mitochondrial malfunctions play a distinct role in AD pathogenesis as well as Aβ and tau aggregates. Immunohistochemistry was applied to study mitochondrial functions: biogenesis, permeability, and motility (fission and fusion), by measuring mitochondrial transcription factor A (TFAM), mitochondrial creatine kinase U-type (CKMT1), and mitochondria fission factor (MFF) protein expression, respectively. The mitochondrial function study showed the ability of THC (0.02 mg/kg dose) to significantly increase MFF protein levels in comparison to vehicle-treated TG mice. Overall, the MFF, TFAM, and CKMT1 protein expression studies did not demonstrate any significant mitochondrial parameter decline in the APP/AS1 mice in comparison to the NTG mice.

### 2.5. Cytokine Profile Changes after the Course of Intranasal THC Treatment

Cytokines are involved in various physiological and pathological pathways; therefore, the alterations in cytokine levels reflect the disturbance of the immune system in AD. The multiplex immunoassay platform was implemented for the cytokine profile study. Within this study, we observed a few Alzheimer’s-related inflammatory molecules: tumor necrosis factor α (TNFα), as the main player in AD pathophysiology [21]; IL-22, and CD40 ligand, which are associated with BBB disruption and neuroinflammation increase [22,23,24]. The effect of THC on plasma cytokine levels was evaluated before and after the intranasal administration of 0.002 and 0.02 mg/kg THC daily for 3 months. The results show no changes in the IL-22, TNFα, or CD40L profiles between the TG and NTG animals. Importantly, the THC treatment results show no effect on cytokine levels in the blood, probably due to the reduced systemic exposure.

### 2.6. The Effect of Low-Dose THC Intranasal Treatment on the Immune System

An immunoglobulin (Ig) isotyping multiplex assay was employed to assess the effect of low-dose THC intranasal treatment on the immunological changes. As shown in Figure 4, IgG1, IgG2a, IgG2b, IgG3, IgA, and IgM levels did not undergo any significant changes after the 0.002 or 0.02 mg/kg THC treatment. Additionally, no evidence of improvement or alteration in immunoglobulin levels was detected. Notably, the three APP/PS1 mouse groups demonstrated steadily lower IgG3 and IgM concentrations in plasma in comparison with the NTG controls. Additionally, the IgA plasma concentration in vehicle-treated TG mice decreased significantly compared to that of the NTG control. In contrast, the obtained data do not indicate any statistically significant difference in IgA levels between the THC-treated TG mice and the NTG controls. Additionally, the level of IgM in blood samples taken from mice after 3 months of 0.02 mg/kg THC treatment slightly improved in comparison to the blood sample data prior to treatment. 

### 2.7. Intranasal THC Treatment Impact on Immune Cell Profiling

The immune system is an integral component of the inflammatory response in the pathophysiology of AD. The aim of this study was to evaluate the differences in immune system parameters in TG mice, in mice treated with the vehicle or THC (0.002 or 0.02 mg/kg dose) daily for 3 months, and in non-treated NTG controls. The relationship between the changes in peripheral blood parameters was investigated using flow cytometry analysis. Three groups of TG mice showed no difference in values compared to the NTG controls, particularly in three parameters: relative and absolute counts of CD3+ naïve T cells; relative and absolute counts of CD3+/CD4+/CD62L+ helper T lymphocytes; and absolute count of CD19+/B220+ resting B cells.

## 3. Discussion

The results of our previous study demonstrated that intraperitoneal injection of THC at 0.02 mg/kg for 3 months significantly improved the spatial learning of aged APP/PS1 mice [14]. In the present study, we evaluated the feasibility and efficacy of chronic low-dose THC treatment via the intranasal route for AD. The behavioral experiments clearly showed that chronic intranasal administration of a low dose of THC slowed down Alzheimer’s-associated cognitive impairments in APP/PS1 mice. The 12-month-old APP/PS1 mice demonstrated an intense decline in memory performance in comparison to the wildtype mice of the same age. Notably, the TG control mice exhibited a constant decline in spatial memory throughout the study, while the APP/PS1 mice treated daily with 0.002 or 0.02 mg/kg THC demonstrated a steady, gradual improvement in cognitive performance. Two mouse groups intranasally treated with THC every day for three months exhibited the same number of errors and latency values as the NTG controls (Figure 2). Overall, the RAWM test demonstrated that three months of low-dose THC intranasal treatment caused a distinctive improvement in cognitive performance and that it can help in memory protection and rescue in AD-related dementia. 

Together with the significant inhibition of AD-related cognitive decline evaluated by RAWM, our study demonstrates that intranasal treatment with a low dose of THC reduced Aβ1–40 and 1–42 peptide levels. The quantitative evaluation of Aβ peptide levels in the hippocampus and cerebral cortex consistently showed that low-dose THC treatment had an inhibitory effect on Aβ production. Specifically, the 0.02 mg/kg THC treatment significantly reduced both soluble Aβ1–40 and insoluble Aβ1–42 deposits in the selected brain regions (Figure 4). Supposedly, the divergent changes in the levels of soluble and insoluble fractions of Aβ1–40 and 1–42 in the brain might reflect a decreasing tendency to favor the production or retention of total Aβ and the formation of aggregates. At the same time, the remarkable decrease in Aβ levels in the brain was associated with elevated Aβ1–40 and Aβ1–42 levels in plasma (Figure 3), implicating that THC increases the clearance of soluble Aβ from the brain into the blood. Actually, the removal of Aβ species from the brain by transport across the BBB is essential to prevent their potentially neurotoxic accumulations in the brain [25]. We assume that such an ability of THC opens up a new pharmacotherapy path for Alzheimer’s and other neurodegenerative diseases. Actually, our findings suggest that low doses of THC do not have any psychotropic effects; consequently, low-dose THC may provide not only an effective but also a safe treatment for cognitive decline and memory impairment in aging and diseased humans. 

**Figure 6:**
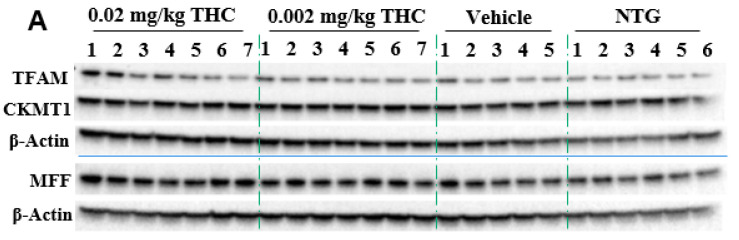

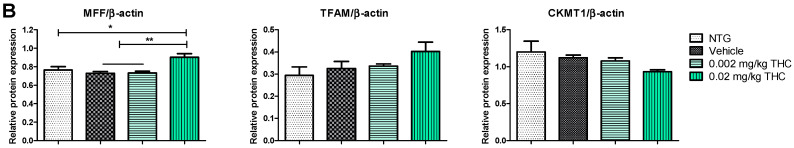
Evaluation of the THC effect on protein expression of MFF, TFAM, and CKMT1 in the brain using Western blot analysis. (**A**) Representative Western blot images of the expression of MFF, TFAM, and CKMT1. (**B**) Relative protein expression levels of MFF, TFAM, and CKMT1 normalized to β-actin. The brain tissue samples were collected from the TG mice after the 3-month THC or vehicle intranasal treatment and from the NTG mice. Densitometric analysis of Western blot results demonstrated a significant difference in the expression of MFF protein in the brains of APP/PS1 mice treated with 0.02 mg/kg THC for 3 months. No statistically significant differences in TFAM and CKMT1 were observed between NTG, vehicle, and THC treatment in APP/PS1 mice. The data are expressed as mean ± SEM (*n* = 5 for the TG vehicle-treated group; *n* = 6 for the NTG group; and *n* = 7 for TG 0.002 and 0.02 mg/kg THC-treated groups). SEM is denoted by error bars. * *p* < 0.05, ** *p* < 0.01 were compared among vehicle-treated TG mice, NTG mice, and APP/PS1 mice treated with 0.002 and 0.02 mg/kg THC using a one-way ANOVA followed by a Tukey post hoc multiple comparison test.

In order to substantiate this significant behavioral improvement and reduction in Aβ aggregates, we checked the neuropathologic parameters within the brain. Specifically, the 3-month course administration of 0.002 and 0.02 mg/kg of THC resulted in a dose-dependent reduction (up to 12.6% and 45.2%, respectively) in the p-tau protein content in the brain in comparison to the TG controls (Figure 5), with no significant influence on GSK3β and p-GSK3β. THC’s ability to decrease the p-tau level is beneficial as tau influences, directly and indirectly, the mitochondrial transport along the neuronal axon, and mitochondrial functions [26,27]. Even though mitochondrial dysfunction has been indicated as an underlying mechanism of AD pathophysiology, no mitochondrial marker impairments within the TG APP/PS1 control group were detected. Additionally, we found that continuous intranasal treatment with 0.02 mg/kg of THC could stimulate (up to 23.7%) MFF expression (Figure 6), which demonstrates an increase in fission. Thus, our future studies will be directed at the impact of THC on fusion markers in order to test if mitochondrial dynamics are in balance.

**Figure 7:**
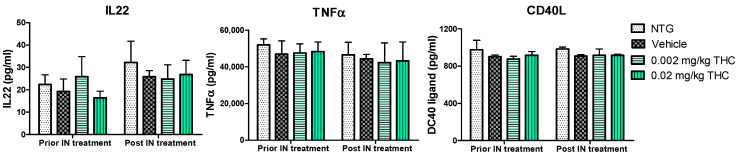
Evaluation of the THC effect on plasma cytokine levels. A control group of APP/PS1 mice underwent intranasal (IN) treatment of vehicles while the experimental groups received 0.002 mg/kg THC or 0.02 mg/kg THC daily for 3 months. Plasma cytokine levels were determined using ELISA before and after treatments were completed. Data are presented as mean ± SD (*n* = 3 per group). Statistical analysis was conducted using a two-way repeated measures ANOVA multiple comparison test. No statistically significant difference in cytokine levels was found among the study groups.

**Figure 8:**
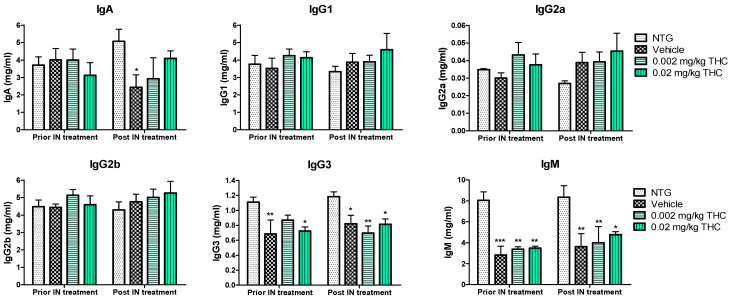
Estimation of immunoglobulin changes after 3 months of intranasal (IN) treatment with THC or vehicle using ELISA. Blood samples were collected from the NTG and TG mice prior to and post-3-month THC or vehicle intranasal treatment. No significant difference in IgG1, IgG2a, IgG2b, IgG3, IgA, and IgM levels was found before and after intranasal treatment. Data are expressed as mean ± SEM (*n* = 5 per group). No statistically significant difference in plasma IgG1, IgG2a, or IgG2b levels was observed between any of the study groups. SEM is denoted by error bars. * *p* < 0.05, ** *p* < 0.01, and *** *p* < 0.001 were compared among NTG mice, vehicle-treated APP/PS1 mice, and APP/PS1 mice treated with 0.002 and 0.02 mg/kg THC using a two-way repeated measures ANOVA multiple comparison test.

**Figure 9:**
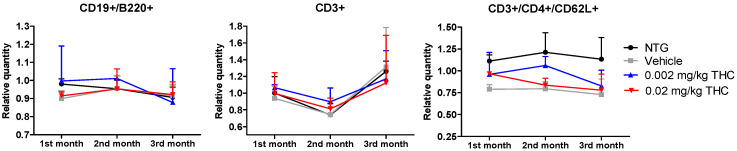
Determination of THC intranasal treatment effect on the immune cell profile. A control group of APP/PS1 mice was given intranasal administration of vehicles while the experimental groups received 0.002 mg/kg THC or 0.02 mg/kg THC daily for 3 months. Immune cell phenotyping was conducted using FACS analysis prior to and post treatment. Data are expressed as mean ± SD (*n* = 3 per group). Statistical analysis was conducted using a 2-way repeated measures ANOVA. No statistically significant difference in the immune cell profile was found among the study groups.

Furthermore, we hypothesized that the amelioration in cognitive performance and biochemical characteristics might be accompanied by immune system changes. Therefore, the cytokine changes were studied, as the primary function of cytokines is to regulate inflammation; as such, they play a vital role in regulating the immune response in health and disease. The evaluation of cytokine plasma levels as a result of nasal treatment with THC demonstrated no changes in the cytokine profile (Figure 7). Further, since the concentrations of cytokines in plasma did not reflect any difference in the inflammatory events between the TG and NTG mice, we further studied the other parameters of the immune system. A further study showed a significant decrease in the expression of IgA, IgG3, and IgM in the AD model mice in comparison to the NTG animals (Figure 8). Interestingly, continuous THC treatment had a positive impact on IgA and IgM levels, which demonstrates the activation of an adaptive immune response. At the same time, no effect of THC treatment on immunoglobulin G-type levels was detected. Immune cell biomarker analysis showed no evidence of an increase or alteration in surface marker expression between the vehicle-treated or THC-treated TG mice and NTG mice (Figure 9). Although intranasal THC administration allows avoiding the first-pass metabolism and THC degradation in the body, not much evidence of THC peripheral action was detected. Interestingly, with the studies presented in Figure 7 and Figure 9, we did not find any signs of peripheral immune system decline or systemic inflammation development related to AD progression in the APP/PS1 mice. Further studies of intranasal THC treatment of AD are needed since these findings do not reflect neuroinflammatory, neurodegeneration, and neurogenesis processes. At the same time, we can conclude that intranasal THC administration is beneficial as it provides non-invasive brain targeting bypassing the BBB, thus enhancing both the bioavailability and absorption speed of THC, reducing systemic exposure and thus unwanted systemic side effects.

## 4. Materials and Methods

### 4.1. Animals and Drug Preparation

**Figure 10:**
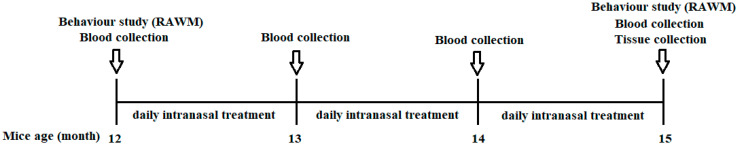
Schematic illustration of the animal testing timeline. NTG control mice and TG mice (12 months of age) were divided into four groups: (1) NTG control; (2) APP/PS1 TG control; (3) APP/PS1—0.002 mg/kg THC treatment; and (4) APP/PS1—0.02 mg/kg THC treatment. Prior to the start of any treatment, groups were balanced with respect to gender, plasma Aβ level, body weight, and baseline memory test results within gender. TG animals were administered intranasal treatment of the vehicle or THC daily for 3 months. Individual animals were subjected to the radial arm water maze test before and after the treatment started. Plasma samples prepared from whole blood were collected monthly and stored at −80 °C. All the animals were euthanized after completing the treatment. Brain tissue samples were collected, and each region was immediately frozen in liquid nitrogen and stored at −80 °C.

Animals: Double transgenic APP_swe_/PS1_ΔE9_ (TG) and C57BL/6J control mice were used in this study, and the number of mice in each group was no fewer than five. APP_swe_/PS1_ΔE9_ mice with a C57BL/6J background were originally purchased from JAX MMRRC (Stock# 034829) and bred in our facility. All APP/PS1 mice were initially genotyped by PCR at the time of grouping. The PCR result was further confirmed by using blood Aβ1–40 measurements. Only the mice expressing Aβ1–40 were used as transgenic mice. The pre-behavior results of the RAWM (error and latency) and the plasma Aβ1–40 levels were balanced among the TG groups (Figure 10). 

All procedures with animals were conducted in compliance with the National Institutes of Health Guide for the Care and Use of Laboratory Animals. The APP/PS1 transgenic mice and non-transgenic control mice were maintained at the University of South Florida Department of Comparative Medicine’s pathogen-free animal facility. All mice were acclimated in our vivarium at 20–26 °C at 30–70% humidity with a 12 h light/dark cycle and were held as one mouse per cage. All animal experiments were approved by the Institutional Animal Care and Use Committee (IACUC) (Project ID: IS00000959) and performed according to the National Institutes of Health (NIH) guidelines.

Drug preparation: THC (CAS No. 1972-08-3, lot D9101515-01) was manufactured by Austin Pharma, LLC (Round Rock, TX, USA). THC working solutions were dissolved from a stock solution made in ethanol in a vehicle solution consisting of saline. The THC stock solution with a concentration of 7.5 µg/µL was prepared in ethanol and stored in a −20 °C freezer. Lyophilized Aβ1–42 peptide (Catalog No.: 1409-rPEP-02. Biomer Technology, Pleasanton, CA, USA) was suspended in pre-chilled 1,1,1,3,3,3-Hexafluoro-2-propanol (HFIP) on ice to produce 1 mM of the Aβ solution at a concentration of 1 mM. The Aβ solution was kept stirred at RT for 24 h until completely dissolved. The Aβ solution was then aliquoted at 10 µL into pre-chilled tubes and centrifuged at 1000× *g* using Speedvac to evaporate the HFIP. The dried solutes were stored at −80 °C. Before use, Aβ was reconstituted in 1% NH_4_OH to 10 mg/mL and diluted into a working solution with 1xTris-buffered saline (TBS). All other chemicals and solvents were obtained from commercial sources. 

### 4.2. Intranasal Treatment

THC was administered intranasally at doses of 0.002 or 0.02 mg/kg. Intranasal administration was performed daily for 3 months on mice lightly anesthetized with isoflurane. For intranasal administration, we applied standard methods of instillation accepted elsewhere [28]. Each mouse was gently grasped by the back of the neck with the abdomen facing upwards while 6 μL of solution volume was instilled in a nostril dropwise over 30 s with a 2 min break between each pair of instillations. 

Prior to the start of any treatment, groups were constituted according to the following criteria in order to minimize between-group variability. Groups were balanced with respect to gender, plasma Aβ levels, body weight, and baseline memory test results varied within gender. 

All experiments were performed on 25 mice. A total of 19 middle-aged transgenic APP/PS1 mice were used for behavioral experiments, as well as 6 non-transgenic mice. NTG control mice and APP/PS1 TG mice were divided into four groups: (1) NTG control; (2) APP/PS1 vehicle-treated TG control; (3) APP/PS1—0.002 mg/kg THC treatment; and (4) APP/PS1—0.02 mg/kg THC treatment. Some mouse samples were collected before they were required to be sacrificed. Thus, all behavior results included 5 mice per group.

### 4.3. Radial Arm Water Maze Behavior Study

Behavioral assays were carried out during the dark phase before and after treatment with THC or the vehicle. Each group of mice (*n* = 5 for each treatment) was subjected to a series of behavioral assays. 

The radial arm water maze test was performed to evaluate the effect of THC on the spatial learning and memory of APP/PS1 transgenic mice by a method described elsewhere [29,30]. Six dividers were placed into a 100 cm circular pool emanated from the center to create a six-arm water maze. The submerged clear platform was placed at the end of one of the six swim arms. The platform location was changed daily to a different arm in a semi-random pattern, and different start arms for each of the daily trials (5 trials) were selected from the remaining five swim arms in a semi-random sequence that involved all arms. On any given day of testing, four acquisition trials and one retention trial after a 30 min delay were conducted. For any given trial, the number of arm selection errors (errors) and the escape latency time (latency) prior to escaping onto the platform were recorded. For each trial, the tested mouse was placed into the start arm facing the center of the pool and given 60 s to find the platform with a 30 s stay. Each time the mouse entered, a non-platform-containing arm would pull back gently into the start arm and an error was recorded. An error was also recorded if the mouse failed to enter any arm within 15 s, or if a mouse entered the platform containing the arm but was unable to find the platform. The mouse was guided to the platform if it failed to find the platform within any 60 s trial and was allowed to stay on the platform for 30 s. A failed platform-finding trial was assigned a latency of 60 s. One extra error was recorded when the mouse refused to make at least three choices during the trial. The test period for each mouse was 9–15 days, depending on performance. Prior to the beginning of THC treatment, every mouse was tested on the RAWM device, with five trials each day for 9 days. For the evaluation of post-treatment spatial learning and memory, individual mice were tested for 15 days. Data of 9 or 15 days of study were grouped into three or five blocks, respectively (every 3 days of data as one block). The first few blocks represent animal familiarization with the experimental procedure, where mice were trained to understand that finding a platform is their ultimate goal. Effective analysis of the experiment started with block 3. Statistical analysis using ANOVA was performed for the averaged data of blocks. 

### 4.4. Sample Collection and Tissue Preparation

Blood was collected before treatment and monthly during treatment from the submandibular vein with an EDTA tube. Tubes were kept on ice and centrifuged at 300× *g* for 5 min; serum samples were then immediately separated and stored at −80 °C. Every animal underwent heart perfusion with 25 mL of normal saline. On the day following the last behavioral assay, mice were anesthetized with SomnaSol (Henry Schein Animal Health, Cat#024352) and intracardially perfused with 50 mL of saline. Blood samples were taken by intracardial assay, the brain was removed via a sagittal bisection, and the left half was immersed in freshly prepared 4% paraformaldehyde in PBS (pH 7.4) for histopathology. The rostral portion of this half was processed for biochemical analysis. The right half was dissected into the hippocampus (HP) and cerebral cortex (cCX). Each region was immediately frozen in liquid nitrogen and stored at −80 °C.

### 4.5. Protein Extraction

On the day of the final brain tissue preparation, frozen tissue was thawed and homogenized in RIPA buffer containing proteinase inhibitor (100 mM Tris, 150 mM NaCl, 0.5% DOC, 1% nonidet P-40, 0.2% SDS, 1 mM Na_3_VO_4_, 10 mM NaF, 1 mM PMSF, 20 uM Leupeptin) with a pellet pestle motor and 10 s sonication and then centrifuged for 20 min at 21,000× *g* at 4 °C. Crude protein concentrations were determined by Bio-rad DC protein assay (Bio-Rad Cat:5000112) and adjusted to the same level for all the samples. The supernatants obtained from this protocol were stored at −80 °C. The soluble and insoluble Aβ peptide extraction was based on the protocol by Izco M et al., with a slight modification [31]. Guanidine-HCl at a concentration of 6 M was used to dissolve the insoluble Aβ pellet.

### 4.6. Aβ1–40 and Aβ1–42 Peptide Measurement by ELISA Assay

Plasma, hippocampal, and cortical levels of soluble Aβ1–40 and Aβ1–42 were measured using a commercially available Amyloid beta Human ELISA kit (MegaNanoDiagmostics Inc., Tampa, FL, USA) according to the manufacturer’s instructions. Briefly, the brain tissues were homogenized in 400 µL RIPA buffer and sonicated for 20 s on ice. Samples underwent centrifugation, and the supernatants were stored at −80 °C. Plasma Aβ1–40 and Aβ1–42 levels were determined from pre-treatment and during/post-treatment blood samples using the same ELISA kits. For the insoluble Aβ, the pellet after preparation for soluble Aβ was dissolved with guanidine-HCl and diluted with assay buffer to 1:5000 for Aβ measurement with the same assay as the soluble Aβ kits. Brain tissue samples’ Aβ1–40 and Aβ1–42 peptide concentrations are presented in ng/mg of total protein. Plasma Aβ1–40 and Aβ1–42 peptide concentrations are presented in pg/mL of plasma. Wavelength readings were performed using a Synergy H1 Hybrid Multi-Mode Reader (BioTek Instruments, Winooski, VT, USA) and corrected by subtracting the readings at 540 nm from the readings at 450 nm. Aβ was quantified using standard curves of the synthetic peptides Aβ1–40 and Aβ1–42. 

### 4.7. Western Blot Detection for Protein Expression 

Immunoblotting was carried out with the following primary antibodies: GSK3β, p-GSK3β, tau, p-tau, MFF, TFAM, CKMT1, and *β*-actin. 

Equal amounts of mouse brain protein samples were denatured with a loading buffer (Invitrogen Cat: NP0007) containing 6% β-mercaptoethanol and heated at 70 °C for 10 min. An equal amount of total proteins (20 µg/well) was then loaded into each well and separated using a 10% Bis-Tris gel. Precision plus protein dual color standards (Bio-rad, #1610374) were used as molecular standards. The separated samples were transferred with a wet assay to a PVDF membrane (Millipore Cat: IPFL00010). Membranes were first blocked with 0.2% Iblock buffer for 1 h at room temperature and then incubated with the primary antibody at designated dilutions in blocking buffer on a shaker overnight at 4 °C. After washing with 1xPBST three times for 5 min, blots were incubated with the appropriate horseradish peroxidase-conjugated secondary antibody in a blocking buffer for 1 h. The enhanced chemiluminescence substrate (Thermo Scientific Prod #34078) was used to develop the blots. Image J software was used for gel quantification. Primary antibodies used for the protein detection were: p-GSK3β (Cell signaling Cat: 9336S) (1:2000); GSK3β (Cell signaling Cat: 9315S) (1:2000); p-tau 217 and tau (MegaNano Diagnostics Inc.) (1:3000); TFAM (Biolegend #850501) (1:2000); MFF (Biolegend # 857501) (1:4000); CKMT1 (Biolegend #867201) (1:4000); β-Actin (Sigma Cat: A5441) (1:10,000).

### 4.8. Plasma Cytokines 

Cytokine expression levels in plasma were determined using the Luminex xMAP technologies Th1/Th2 Cytokine 11-Plex Mouse ProcartaPlex™ Panel (Catalog #: EPX110-20820-901, Thermo Fisher Scientific, Waltham, MA, USA). The mouse cytokine/chemokine magnetic bead panel was used for IL-22 and TNFα detection according to the manufacturer’s protocol. In brief, plasma samples and standards were diluted with sample dilution buffer and mixed with magnetic beads and then the detection antibody. Immediately after the wash, the plate was read on a Bio-Plex^®^ MAGPIX^TM^ Multiplex Reader (Bio-Rad Laboratories, Inc., Hercules, CA, USA). The concentration of each cytokine in each sample was calculated using the standard curve of each cytokine. The CD40L concentration in plasma was measured using a commercially available Mouse CD40L ELISA kit (Catalog #: ab119517, Abcam, Waltham, MA, USA) according to the manufacturer’s instructions. Wavelength readings were performed using a Synergy H1 Hybrid Multi-Mode Reader (BioTek Instruments, Winooski, VT, USA) at 450 nm. 

### 4.9. Immunoglobulin Isotyping 

Antibody Ig isotyping was performed using a magnetic bead-based multiplex assay (Millipore Cat: MGAMMAG-300K). The assay was performed according to the manufacturer’s instructions. The results were obtained by reading the plates on the Bioplex MAGPIX Multiplex Reader, and the concentration was calculated by using Bio-plex manager 6.1 software. A total of 6 markers (IgA, IgG1, IgG2a, IgG2b, IgG3, and IgM) were tested and quantified for each standard.

### 4.10. Immunophenotyping by Flow Cytometry Analysis

Blood samples were collected from the mandibular vein into sample tubes pre-coated with EDTA anticoagulant. For the surface staining of white blood cells, 50 µL of blood was added to tubes containing 5 µL of fluorochrome-labeled monoclonal antibodies. 

Blood samples were incubated with ACK (Ammonium-Chloride-Potassium) Lysing buffer to lyse the RBC for 1 min and washed with 1XPBS twice, and antibodies were added and incubated for 15 min at room temperature in the dark. Flow cytometric evaluation was conducted with a Guava flow cytometer (Millipore Guava EasyCyte 8HT Flow Cytometer). A minimum of 10,000 events were obtained for each stain and were supplied in list mode. Multiple peripheral blood parameters were assessed as absolute and relative values. The gating strategies for the different lymphocyte subsets assessed were as follows: T lymphocytes (CD3+); B lymphocytes (CD19+/B220+); naïve helper T lymphocytes (CD3+/CD4+/CD62L+). The absolute values were calculated from the blood counts, and the relative values were calculated as the percentage of the population described. The data were normalized to the control and are presented as the relative quantity. All methods were performed in accordance with the relevant guidelines and regulations [32].

### 4.11. Data Analysis

GraphPad Prism 5.00 software (GraphPad) was used to compare the differences among groups. All results are presented as the mean ± standard error of the mean. Comparison of means between more than two independent groups was conducted using one-way ANOVA followed by Tukey’s post hoc multiple comparison test. A one-way ANOVA with repeated measures statistical analysis was applied to compare changes in mean scores overtime points. Comparison of means between more than three groups was conducted using a regular two-way ANOVA followed by multiple comparison tests. In the case of the use of two-way repeated measures ANOVA multiple comparison test, the *p*-values were adjusted using the Bonferroni multiple testing correction method.

## Data Availability

The data presented in this study are available on request from the corresponding authors.
